# 2-Methyl-6-(4,4,10,13,14-penta­methyl-3-oxo-2,3,4,5,6,7,10,11,12,13,14,15,16,17-tetra­deca­hydro-1*H*-cyclo­penta­[*a*]phenanthren-17-yl)hept-2-enoic acid

**DOI:** 10.1107/S1600536811006283

**Published:** 2011-02-26

**Authors:** Mohammad Arfan, Abdur Rauf, M. Nawaz Tahir, Mumtaz Ali, Ghias Uddin

**Affiliations:** a Institute of Chemical Sciences, University of Peshawar, Peshawar, Pakistan; b Department of Physics, University of Sargodha, Sargodha, Pakistan; c Department of Chemistry, University of Malalkand, KPK, Pakistan

## Abstract

In the title compound, C_30_H_46_O_3_, an isolation product of *Pistacia integerima Stewart*, the five-membered ring is nearly in the envelope form. A 6-carb­oxy­hept-5-en-2-yl group is attached to the five-membered ring. An *S*(6) ring motif is formed due to intra­molecular C—H⋯O hydrogen bonding. In the crystal, inter­molecular O—H⋯O hydrogen bonds form carboxyl­ate dimers with *R*_2_^2^(8) ring motifs.

## Related literature

For related structures and background, see: Lanfredi *et al.* (1975 ▶); Ahmad *et al.* (2010 ▶). For graph-set notation, see: Bernstein *et al.* (1995 ▶). For puckering parameters, see: Cremer & Pople (1975 ▶).
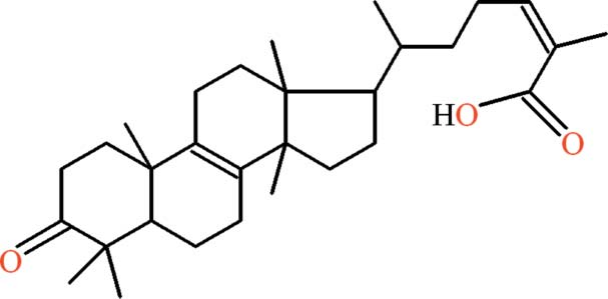


## Experimental

### 

#### Crystal data

C_30_H_46_O_3_
*M* *_r_* = 454.67
Tetragonal, 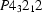

*a* = 13.3167 (6) Å
*c* = 31.1595 (14) Å
*V* = 5525.7 (4) Å^3^
*Z* = 8
Mo *K*α radiation
μ = 0.07 mm^−1^
*T* = 296 K
0.28 × 0.15 × 0.10 mm

#### Data collection

Bruker Kappa APEXII CCD diffractometer
Absorption correction: multi-scan (*SADABS*; Bruker, 2005 ▶) *T*_min_ = 0.935, *T*_max_ = 0.965
80888 measured reflections
3012 independent reflections
1936 reflections with *I* > 2σ(*I*)
*R*_int_ = 0.103

#### Refinement

*R*[*F* ^2^ > 2σ(*F* ^2^)] = 0.061
*wR*(*F* ^2^) = 0.186
*S* = 1.03
3012 reflections
306 parameters
H-atom parameters constrained
Δρ_max_ = 0.20 e Å^−3^
Δρ_min_ = −0.15 e Å^−3^

### 

Data collection: *APEX2* (Bruker, 2009 ▶); cell refinement: *SAINT* (Bruker, 2009 ▶); data reduction: *SAINT*; program(s) used to solve structure: *SHELXS97* (Sheldrick, 2008 ▶); program(s) used to refine structure: *SHELXL97* (Sheldrick, 2008 ▶); molecular graphics: *ORTEP-3 for Windows* (Farrugia, 1997 ▶) and *PLATON* (Spek, 2009 ▶); software used to prepare material for publication: *WinGX* (Farrugia, 1999 ▶) and *PLATON*.

## Supplementary Material

Crystal structure: contains datablocks global, I. DOI: 10.1107/S1600536811006283/bq2281sup1.cif

Structure factors: contains datablocks I. DOI: 10.1107/S1600536811006283/bq2281Isup2.hkl

Additional supplementary materials:  crystallographic information; 3D view; checkCIF report

## Figures and Tables

**Table 1:** Hydrogen-bond geometry (Å, °)

*D*—H⋯*A*	*D*—H	H⋯*A*	*D*⋯*A*	*D*—H⋯*A*
O2—H2⋯O3^i^	0.82	1.85	2.645 (7)	162
C20—H20*A*⋯O2	0.97	2.21	2.873 (7)	125

Symmetry code: (i) 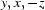.

**Fig. 1.**
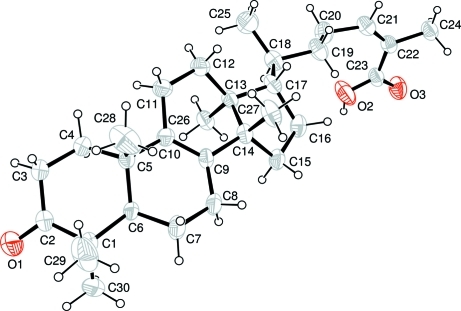
View of the title compound with the atom numbering scheme. The displacement ellipsoids are drawn at the 30% probability level.

**Fig. 2.**
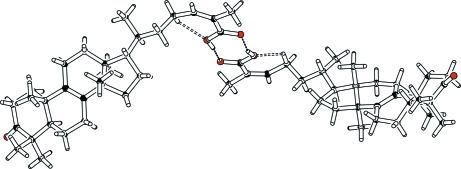
The partial packing (PLATON; Spek, 2009) which shows that molecules form carboxylate dimers with R22(8) ring motif.

## supplementary crystallographic information

### Comment

The crystal structure of Methyl (13α,14β,20*S*,24*Z*)-3-oxo-lanosta-8,24-dien-26-oate (Lanfredi *et al.*, 1975) has been previously published which is closely related to the title compound (I. Fig. 1). This compound has been derived from the berries of *Schinus molle*. The title compound is isolated from the galls of *Pistacia integerima Stewart* which were collected from Razagran, District Dir, KPK, Pakistan. The isolated compound from the galls of *Pistacia integerima Stewart* by Ahmad *et al.*, 2010 and reported as Pisticialanstenoic acid, seems the same as (I). However their spectral studies differ a litle bit from our X-ray analysis.

In (I), three six membered rings A (C1—C6), B (C5—C10) and C (C9—C14) are confirmed by different puckering parameters (Cremer & Pople, 1975). The puckering amplitude Q for the rings A, B and C have values of 0.490 (5) Å, 0.503 (4) Å and 0.540 (4) Å, θ for the rings A, B and C have values of 20.4 (6)°, 131.6 (6)° and 119.3 (4)°, φ for the rings A, B and C have values of 217.0 (17)°, 244.0 (7)° and 69.7 (5)°, respectively. The five membered ring D (C13—C17) has approximately envelop confirmation. The linear chain E (C20/C21/C22/C24) is planar with r. m. s. deviation of 0.0175 Å. The groups F (C18/C19/C25) and G (C23/O2/O3) are of course planar. The dihedral angle between E/F, E/G and F/G is 46.67 (5)°, 8.64 (1)° and 49.61 (5)°, respectively. There exist a strong intramolecular C—H···O and intermolecular O—H···O type H-bonding (Table 1, Fig. 2). Due to the inramolecular H-bonding S(6) ring motif is formed (Bernstein *et al.*, 1995). The molecules are stabilized in the form of conventional carboxylate dimers with *R*_2_^2^(8) ring motifs (Fig. 2).

### Experimental

The dried and crushed galls of Pistacia chinensis var integerima (5 kg) were subjected to cold extraction with methanol (MeOH). The MeOH extract (400 g) was suspended in water and successively partitioned with n-hexane, CHCl_3_, EtOAc and butanol (BuOH). The CHCl_3_ fraction (10 g) was subjected to column chromatography on silica gel. The column was first eluted with n-hexane:EtOAc (100:0 → 0:100) as solvent system. A total of 13 fractions, RF-1 to RF-13 were obtained based on TLC profiles. White crude product of fraction RF-4 was separated from the solution by decantation. This crude material was washed with acetone for several times. White crystals were obtained by re-crystallization from a mixture of n-hexane:acetone (80:20). Yield: 90 mg.

### Refinement

In the absence of anomalous scattering factor all of the Friedel pairs were merged. Initially all H-atoms were taken from the difference Fourier map and at the last stage these H-atoms were geometrically treated.

The H-atoms were positioned geometrically (O—H = 0.82, C–H = 0.93–0.97 Å) and refined as riding with *U*_iso_(H) = x*U*_eq_(C, O), where x = 1.5 for methyl and x = 1.2 for all other H-atoms.

### Crystal data

**Table:**

C_30_H_46_O_3_	*D*_x_ = 1.093 Mg m^−^^3^
*M**_r_* = 454.67	Mo *K*α radiation, λ = 0.71073 Å
Tetragonal, *P*4_3_2_1_2	Cell parameters from 1936 reflections
Hall symbol: P 4nw 2abw	θ = 2.0–25.5°
*a* = 13.3167 (6) Å	µ = 0.07 mm^−^^1^
*c* = 31.1595 (14) Å	*T* = 296 K
*V* = 5525.7 (4) Å^3^	Needle, brown
*Z* = 8	0.28 × 0.15 × 0.10 mm
*F*(000) = 2000	

### Data collection

**Table:**

Bruker Kappa APEXII CCD diffractometer	3012 independent reflections
Radiation source: fine-focus sealed tube	1936 reflections with *I* > 2σ(*I*)
graphite	*R*_int_ = 0.103
Detector resolution: 7.50 pixels mm^-1^	θ_max_ = 25.5°, θ_min_ = 2.0°
ω scans	*h* = −15→15
Absorption correction: multi-scan (*SADABS*; Bruker, 2005)	*k* = −15→15
*T*_min_ = 0.935, *T*_max_ = 0.965	*l* = −36→37
80888 measured reflections	

### Refinement

**Table:**

Refinement on *F*^2^	Primary atom site location: structure-invariant direct methods
Least-squares matrix: full	Secondary atom site location: difference Fourier map
*R*[*F*^2^ > 2σ(*F*^2^)] = 0.061	Hydrogen site location: inferred from neighbouring sites
*wR*(*F*^2^) = 0.186	H-atom parameters constrained
*S* = 1.03	*w* = 1/[σ^2^(*F*_o_^2^) + (0.086*P*)^2^ + 1.2062*P*] where *P* = (*F*_o_^2^ + 2*F*_c_^2^)/3
3012 reflections	(Δ/σ)_max_ < 0.001
306 parameters	Δρ_max_ = 0.20 e Å^−^^3^
0 restraints	Δρ_min_ = −0.15 e Å^−^^3^

### Special details

**Table:**

Geometry. Bond distances, angles *etc*. have been calculated using the rounded fractional coordinates. All su's are estimated from the variances of the (full) variance-covariance matrix. The cell e.s.d.'s are taken into account in the estimation of distances, angles and torsion angles
Refinement. Refinement of *F*^2^ against ALL reflections. The weighted *R*-factor *wR* and goodness of fit *S* are based on *F*^2^, conventional *R*-factors *R* are based on *F*, with *F* set to zero for negative *F*^2^. The threshold expression of *F*^2^ > σ(*F*^2^) is used only for calculating *R*-factors(gt) *etc*. and is not relevant to the choice of reflections for refinement. *R*-factors based on *F*^2^ are statistically about twice as large as those based on *F*, and *R*- factors based on ALL data will be even larger.

### Fractional atomic coordinates and isotropic or equivalent isotropic displacement parameters (Å^2^)

**Table:**

	*x*	*y*	*z*	*U*_iso_*/*U*_eq_	
O1	0.0467 (3)	0.2309 (4)	0.45862 (16)	0.153 (3)	
O2	0.4258 (4)	0.3086 (4)	0.04257 (10)	0.126 (2)	
O3	0.4457 (3)	0.2870 (3)	−0.02665 (10)	0.0945 (15)	
C1	0.0545 (3)	0.1086 (4)	0.40354 (12)	0.0697 (16)	
C2	0.0997 (4)	0.1819 (4)	0.43511 (15)	0.0853 (19)	
C3	0.2098 (4)	0.1923 (6)	0.43906 (16)	0.113 (3)	
C4	0.2651 (3)	0.1886 (4)	0.39648 (13)	0.0790 (19)	
C5	0.2386 (3)	0.0961 (3)	0.36929 (11)	0.0543 (11)	
C6	0.1235 (3)	0.0989 (3)	0.36259 (11)	0.0531 (13)	
C7	0.0906 (4)	0.0152 (4)	0.33306 (13)	0.090 (2)	
C8	0.1410 (3)	0.0268 (4)	0.28990 (12)	0.0683 (16)	
C9	0.2428 (3)	0.0658 (3)	0.28939 (10)	0.0519 (11)	
C10	0.2894 (3)	0.1031 (4)	0.32563 (11)	0.0623 (14)	
C11	0.4001 (3)	0.1270 (4)	0.32455 (11)	0.0750 (18)	
C12	0.4550 (3)	0.1281 (4)	0.28202 (11)	0.0690 (18)	
C13	0.3835 (3)	0.1393 (3)	0.24358 (11)	0.0512 (11)	
C14	0.3017 (3)	0.0559 (3)	0.24792 (11)	0.0545 (14)	
C15	0.2439 (3)	0.0683 (4)	0.20556 (11)	0.0664 (15)	
C16	0.3252 (4)	0.0930 (4)	0.17256 (12)	0.0714 (15)	
C17	0.4235 (3)	0.1184 (3)	0.19773 (11)	0.0587 (14)	
C18	0.4879 (3)	0.1963 (3)	0.17434 (12)	0.0647 (16)	
C19	0.5185 (4)	0.1526 (3)	0.13015 (12)	0.0750 (16)	
C20	0.5711 (4)	0.2247 (4)	0.10054 (12)	0.0767 (18)	
C21	0.5919 (3)	0.1785 (3)	0.05758 (13)	0.0670 (17)	
C22	0.5515 (3)	0.1957 (3)	0.01936 (12)	0.0597 (14)	
C23	0.4697 (3)	0.2684 (3)	0.01166 (14)	0.0667 (17)	
C24	0.5897 (3)	0.1425 (3)	−0.02023 (13)	0.0737 (17)	
C25	0.5801 (4)	0.2295 (4)	0.19933 (15)	0.094 (2)	
C26	0.3361 (3)	0.2445 (3)	0.24457 (13)	0.0650 (16)	
C27	0.3455 (4)	−0.0514 (3)	0.24816 (14)	0.0810 (19)	
C28	0.2784 (5)	0.0019 (5)	0.39218 (17)	0.112 (3)	
C29	0.0378 (6)	0.0107 (5)	0.42829 (18)	0.130 (3)	
C30	−0.0470 (4)	0.1500 (6)	0.38967 (15)	0.111 (3)	
H2	0.37831	0.34194	0.03369	0.1506*	
H3A	0.23489	0.13882	0.45730	0.1358*	
H3B	0.22476	0.25557	0.45307	0.1358*	
H4A	0.33683	0.18870	0.40194	0.0946*	
H4B	0.24930	0.24873	0.38024	0.0946*	
H6	0.11127	0.16060	0.34627	0.0637*	
H7A	0.01822	0.01725	0.32950	0.1080*	
H7B	0.10831	−0.04913	0.34551	0.1080*	
H8A	0.14170	−0.03851	0.27608	0.0820*	
H8B	0.09968	0.07064	0.27243	0.0820*	
H11A	0.43374	0.07888	0.34294	0.0902*	
H11B	0.40888	0.19258	0.33758	0.0902*	
H12A	0.49270	0.06612	0.27900	0.0831*	
H12B	0.50259	0.18325	0.28186	0.0831*	
H15A	0.20905	0.00683	0.19801	0.0794*	
H15B	0.19532	0.12242	0.20762	0.0794*	
H16A	0.30481	0.14991	0.15518	0.0854*	
H16B	0.33623	0.03599	0.15377	0.0854*	
H17	0.46301	0.05644	0.19923	0.0702*	
H18	0.44620	0.25568	0.16920	0.0777*	
H19A	0.56223	0.09537	0.13492	0.0898*	
H19B	0.45857	0.12811	0.11589	0.0898*	
H20A	0.52964	0.28396	0.09671	0.0920*	
H20B	0.63392	0.24570	0.11349	0.0920*	
H21	0.64161	0.12939	0.05775	0.0805*	
H24A	0.63824	0.09279	−0.01204	0.1105*	
H24B	0.62052	0.19041	−0.03912	0.1105*	
H24C	0.53461	0.11059	−0.03468	0.1105*	
H25A	0.62046	0.27268	0.18170	0.1404*	
H25B	0.61863	0.17158	0.20747	0.1404*	
H25C	0.55949	0.26512	0.22462	0.1404*	
H26A	0.29602	0.25408	0.21928	0.0973*	
H26B	0.38814	0.29424	0.24548	0.0973*	
H26C	0.29438	0.25074	0.26957	0.0973*	
H27A	0.38542	−0.06134	0.22286	0.1215*	
H27B	0.29170	−0.09931	0.24862	0.1215*	
H27C	0.38672	−0.06017	0.27316	0.1215*	
H28A	0.25466	−0.05697	0.37756	0.1687*	
H28B	0.25480	0.00123	0.42129	0.1687*	
H28C	0.35044	0.00259	0.39196	0.1687*	
H29A	0.01134	−0.03938	0.40925	0.1956*	
H29B	−0.00884	0.02219	0.45125	0.1956*	
H29C	0.10060	−0.01211	0.43991	0.1956*	
H30A	−0.07966	0.10242	0.37124	0.1662*	
H30B	−0.03736	0.21201	0.37448	0.1662*	
H30C	−0.08784	0.16178	0.41454	0.1662*	

### Atomic displacement parameters (Å^2^)

**Table:**

	*U*^11^	*U*^22^	*U*^33^	*U*^12^	*U*^13^	*U*^23^
O1	0.090 (3)	0.218 (6)	0.152 (4)	−0.011 (3)	0.022 (3)	−0.121 (4)
O2	0.133 (4)	0.169 (4)	0.075 (2)	0.079 (3)	0.009 (2)	−0.017 (3)
O3	0.122 (3)	0.100 (3)	0.0614 (18)	0.021 (2)	0.0003 (19)	−0.0075 (18)
C1	0.066 (3)	0.098 (3)	0.045 (2)	−0.016 (3)	0.010 (2)	−0.017 (2)
C2	0.068 (3)	0.123 (4)	0.065 (3)	−0.005 (3)	0.007 (2)	−0.037 (3)
C3	0.067 (3)	0.191 (7)	0.082 (3)	−0.007 (4)	0.001 (3)	−0.073 (4)
C4	0.059 (3)	0.109 (4)	0.069 (3)	−0.007 (3)	−0.003 (2)	−0.036 (3)
C5	0.063 (2)	0.061 (2)	0.0389 (17)	0.006 (2)	−0.0009 (18)	−0.0028 (18)
C6	0.052 (2)	0.070 (3)	0.0373 (17)	−0.009 (2)	0.0011 (16)	−0.0099 (18)
C7	0.094 (4)	0.116 (4)	0.061 (2)	−0.042 (3)	0.019 (3)	−0.036 (3)
C8	0.075 (3)	0.080 (3)	0.050 (2)	−0.013 (2)	0.004 (2)	−0.022 (2)
C9	0.064 (2)	0.053 (2)	0.0388 (18)	−0.0026 (19)	0.0047 (18)	−0.0079 (17)
C10	0.053 (2)	0.093 (3)	0.0408 (19)	−0.002 (2)	−0.0015 (18)	−0.004 (2)
C11	0.051 (3)	0.129 (4)	0.045 (2)	0.007 (3)	−0.0021 (19)	0.000 (2)
C12	0.057 (3)	0.101 (4)	0.049 (2)	0.006 (2)	0.002 (2)	0.001 (2)
C13	0.053 (2)	0.058 (2)	0.0425 (19)	0.0116 (18)	0.0040 (17)	0.0011 (17)
C14	0.068 (3)	0.051 (2)	0.0445 (19)	0.0072 (19)	0.0025 (19)	−0.0040 (18)
C15	0.076 (3)	0.080 (3)	0.0431 (19)	−0.006 (2)	0.001 (2)	−0.012 (2)
C16	0.092 (3)	0.080 (3)	0.0421 (19)	0.000 (3)	−0.003 (2)	−0.008 (2)
C17	0.067 (3)	0.058 (2)	0.051 (2)	0.018 (2)	0.011 (2)	0.0031 (18)
C18	0.075 (3)	0.058 (3)	0.061 (2)	0.011 (2)	0.011 (2)	0.007 (2)
C19	0.090 (3)	0.073 (3)	0.062 (2)	0.005 (3)	0.027 (2)	0.006 (2)
C20	0.095 (4)	0.078 (3)	0.057 (2)	−0.001 (3)	0.012 (2)	0.004 (2)
C21	0.074 (3)	0.056 (3)	0.071 (3)	0.000 (2)	0.023 (2)	0.008 (2)
C22	0.064 (3)	0.055 (2)	0.060 (2)	−0.008 (2)	0.018 (2)	−0.002 (2)
C23	0.073 (3)	0.064 (3)	0.063 (3)	−0.006 (2)	0.019 (2)	−0.010 (2)
C24	0.075 (3)	0.069 (3)	0.077 (3)	−0.008 (2)	0.024 (2)	−0.014 (2)
C25	0.088 (4)	0.112 (4)	0.081 (3)	−0.016 (3)	0.011 (3)	0.005 (3)
C26	0.069 (3)	0.059 (3)	0.067 (2)	0.005 (2)	0.015 (2)	−0.008 (2)
C27	0.114 (4)	0.060 (3)	0.069 (3)	0.018 (3)	0.023 (3)	0.001 (2)
C28	0.120 (5)	0.110 (5)	0.107 (4)	0.045 (4)	0.025 (4)	0.042 (4)
C29	0.171 (7)	0.131 (5)	0.089 (4)	−0.039 (5)	0.066 (4)	0.004 (4)
C30	0.053 (3)	0.203 (7)	0.077 (3)	−0.010 (4)	0.009 (2)	−0.029 (4)

### Geometric parameters (Å, °)

**Table:**

O1—C2	1.209 (7)	C6—H6	0.9800
O2—C23	1.247 (6)	C7—H7A	0.9700
O3—C23	1.260 (5)	C7—H7B	0.9700
O2—H2	0.8200	C8—H8A	0.9700
C1—C2	1.511 (7)	C8—H8B	0.9700
C1—C6	1.578 (5)	C11—H11A	0.9700
C1—C30	1.522 (7)	C11—H11B	0.9700
C1—C29	1.531 (8)	C12—H12A	0.9700
C2—C3	1.478 (8)	C12—H12B	0.9700
C3—C4	1.518 (6)	C15—H15A	0.9700
C4—C5	1.536 (6)	C15—H15B	0.9700
C5—C6	1.547 (6)	C16—H16A	0.9700
C5—C28	1.537 (7)	C16—H16B	0.9700
C5—C10	1.522 (5)	C17—H17	0.9800
C6—C7	1.510 (6)	C18—H18	0.9800
C7—C8	1.511 (6)	C19—H19A	0.9700
C8—C9	1.452 (6)	C19—H19B	0.9700
C9—C10	1.381 (5)	C20—H20A	0.9700
C9—C14	1.517 (5)	C20—H20B	0.9700
C10—C11	1.509 (6)	C21—H21	0.9300
C11—C12	1.514 (5)	C24—H24A	0.9600
C12—C13	1.537 (5)	C24—H24B	0.9600
C13—C17	1.550 (5)	C24—H24C	0.9600
C13—C26	1.537 (6)	C25—H25A	0.9600
C13—C14	1.562 (6)	C25—H25B	0.9600
C14—C15	1.537 (5)	C25—H25C	0.9600
C14—C27	1.543 (6)	C26—H26A	0.9600
C15—C16	1.529 (6)	C26—H26B	0.9600
C16—C17	1.563 (6)	C26—H26C	0.9600
C17—C18	1.531 (6)	C27—H27A	0.9600
C18—C25	1.520 (6)	C27—H27B	0.9600
C18—C19	1.549 (5)	C27—H27C	0.9600
C19—C20	1.505 (6)	C28—H28A	0.9600
C20—C21	1.499 (6)	C28—H28B	0.9600
C21—C22	1.327 (6)	C28—H28C	0.9600
C22—C23	1.477 (6)	C29—H29A	0.9600
C22—C24	1.511 (6)	C29—H29B	0.9600
C3—H3A	0.9700	C29—H29C	0.9600
C3—H3B	0.9700	C30—H30A	0.9600
C4—H4A	0.9700	C30—H30B	0.9600
C4—H4B	0.9700	C30—H30C	0.9600
			
O2···O3^i^	2.645 (7)	H11B···C4	2.6500
O2···C20	2.873 (7)	H11B···H4A	2.2200
O3···O2^i^	2.645 (7)	H12A···C27	2.6900
O1···H30C	2.4400	H12A···H17	2.5200
O1···H30B	2.8600	H12A···H27C	2.2000
O1···H3A^ii^	2.8900	H12B···C25	2.8400
O1···H29B	2.8900	H12B···H25C	2.2200
O2···H20A	2.2100	H12B···H26B	2.4100
O2···H2^i^	2.7800	H15A···C8	3.0100
O2···H30A^iii^	2.8900	H15A···H27B	2.3900
O3···H24B	2.6900	H15B···C8	2.9500
O3···H25B^iv^	2.7800	H15B···C26	2.7400
O3···H8B^v^	2.8000	H15B···H8B	2.4900
O3···H24C	2.6400	H15B···H26A	2.2400
O3···H2^i^	1.8500	H15B···C21^x^	3.0200
C10···C26	3.211 (6)	H16A···C19	2.9500
C11···C27	3.441 (6)	H16A···C26	3.0900
C12···C25	3.352 (6)	H16A···H18	2.3900
C20···O2	2.873 (7)	H16A···H19B	2.4000
C25···C26	3.548 (7)	H16A···H26A	2.4300
C25···C12	3.352 (6)	H16B···C19	2.9700
C26···C25	3.548 (7)	H16B···H19B	2.3600
C26···C10	3.211 (6)	H16B···H27A	2.6000
C27···C11	3.441 (6)	H16B···C11^iv^	3.1000
C28···C29	3.398 (10)	H16B···H11A^iv^	2.5900
C29···C28	3.398 (10)	H17···C27	2.6100
C1···H28B	3.0800	H17···H12A	2.5200
C3···H29C	3.0900	H17···H19A	2.4600
C3···H28B	2.6700	H17···H25B	2.5900
C4···H11B	2.6500	H17···H27A	2.0200
C7···H29A	2.7000	H17···H24C^vi^	2.3600
C7···H28A	2.7600	H18···C26	2.7700
C7···H30A	2.8100	H18···H16A	2.3900
C8···H27B	2.9200	H18···H20A	2.5500
C8···H15B	2.9500	H18···H26A	2.5400
C8···H15A	3.0100	H18···H26B	2.5500
C9···H6	2.7900	H18···C30^iii^	3.0600
C9···H26C	2.6300	H18···H30C^iii^	2.5400
C10···H26C	2.6300	H19A···H17	2.4600
C10···H27C	3.0100	H19A···H25B	2.5900
C11···H26C	2.7600	H19B···C16	2.5500
C11···H16B^vi^	3.1000	H19B···H16A	2.4000
C11···H27C	2.9700	H19B···H16B	2.3600
C11···H4A	2.6800	H20A···O2	2.2100
C11···H28C	2.7600	H20A···C23	2.7800
C12···H27C	2.6800	H20A···H18	2.5500
C12···H25C	2.9100	H20B···C25	2.7800
C13···H25C	2.9400	H20B···H25A	2.1600
C15···H26A	2.6000	H21···H24A	2.2300
C15···H8A	2.9500	H24A···H21	2.2300
C15···H8B	2.8300	H24B···O3	2.6900
C16···H19B	2.5500	H24B···H26C^v^	2.5200
C16···H27A	2.7100	H24C···O3	2.6400
C16···H26A	2.6200	H24C···H17^iv^	2.3600
C17···H27A	2.5700	H25A···C20	2.6900
C18···H26A	3.0100	H25A···H20B	2.1600
C18···H26B	2.8900	H25B···H17	2.5900
C19···H16A	2.9500	H25B···H19A	2.5900
C19···H16B	2.9700	H25B···O3^vi^	2.7800
C20···H25A	2.6900	H25C···C12	2.9100
C21···H26A^v^	2.9800	H25C···C13	2.9400
C21···H15B^v^	3.0200	H25C···C26	3.0500
C23···H8B^v^	2.9500	H25C···H12B	2.2200
C23···H20A	2.7800	H25C···H26B	2.4000
C23···H2^i^	2.6500	H26A···C15	2.6000
C24···H26C^v^	3.0700	H26A···C16	2.6200
C25···H20B	2.7800	H26A···C18	3.0100
C25···H12B	2.8400	H26A···H15B	2.2400
C25···H26B	3.0600	H26A···H16A	2.4300
C26···H18	2.7700	H26A···H18	2.5400
C26···H15B	2.7400	H26A···C21^x^	2.9800
C26···H16A	3.0900	H26B···C18	2.8900
C26···H25C	3.0500	H26B···C25	3.0600
C27···H17	2.6100	H26B···H12B	2.4100
C27···H8A	2.8600	H26B···H18	2.5500
C27···H12A	2.6900	H26B···H25C	2.4000
C28···H3A	2.7900	H26C···C9	2.6300
C28···H11A	2.7700	H26C···C10	2.6300
C28···H29C	2.8000	H26C···C11	2.7600
C28···H7B	2.7800	H26C···C24^x^	3.0700
C29···H7A	3.0900	H26C···H24B^x^	2.5200
C29···H7B	2.8600	H27A···C16	2.7100
C29···H28B	2.9000	H27A···C17	2.5700
C30···H7A	2.7200	H27A···H16B	2.6000
C30···H18^vii^	3.0600	H27A···H17	2.0200
H2···O2^i^	2.7800	H27B···C8	2.9200
H2···O3^i^	1.8500	H27B···H8A	2.3200
H2···C23^i^	2.6500	H27B···H15A	2.3900
H2···H2^i^	2.2100	H27B···H3B^xi^	2.4600
H3A···C28	2.7900	H27C···C10	3.0100
H3A···H28B	2.1600	H27C···C11	2.9700
H3A···O1^ii^	2.8900	H27C···C12	2.6800
H3B···H27B^viii^	2.4600	H27C···H12A	2.2000
H4A···C11	2.6800	H28A···C7	2.7600
H4A···H11B	2.2200	H28A···H7B	2.1900
H4A···H28C	2.5000	H28A···H4B^xi^	2.6000
H4B···H6	2.4200	H28B···C1	3.0800
H4B···H28A^viii^	2.6000	H28B···C3	2.6700
H6···C9	2.7900	H28B···C29	2.9000
H6···H4B	2.4200	H28B···H3A	2.1600
H6···H8B	2.6000	H28B···H29C	2.1400
H6···H30B	2.2700	H28C···C11	2.7600
H7A···C29	3.0900	H28C···H4A	2.5000
H7A···C30	2.7200	H28C···H11A	2.1400
H7A···H29A	2.6000	H29A···C7	2.7000
H7A···H30A	2.1600	H29A···H7A	2.6000
H7B···C28	2.7800	H29A···H7B	2.3700
H7B···C29	2.8600	H29A···H30A	2.5400
H7B···H28A	2.1900	H29B···O1	2.8900
H7B···H29A	2.3700	H29B···H30C	2.4200
H8A···C15	2.9500	H29C···C3	3.0900
H8A···C27	2.8600	H29C···C28	2.8000
H8A···H27B	2.3200	H29C···H28B	2.1400
H8A···H8A^ix^	2.5300	H30A···C7	2.8100
H8B···C15	2.8300	H30A···H7A	2.1600
H8B···H6	2.6000	H30A···H29A	2.5400
H8B···H15B	2.4900	H30A···O2^vii^	2.8900
H8B···O3^x^	2.8000	H30B···O1	2.8600
H8B···C23^x^	2.9500	H30B···H6	2.2700
H11A···C28	2.7700	H30C···O1	2.4400
H11A···H28C	2.1400	H30C···H29B	2.4200
H11A···H16B^vi^	2.5900	H30C···H18^vii^	2.5400
			
C23—O2—H2	109.00	H8A—C8—H8B	107.00
C2—C1—C29	106.3 (4)	C10—C11—H11A	107.00
C2—C1—C30	107.7 (4)	C10—C11—H11B	107.00
C2—C1—C6	110.3 (4)	C12—C11—H11A	108.00
C6—C1—C30	108.5 (3)	C12—C11—H11B	107.00
C29—C1—C30	108.8 (5)	H11A—C11—H11B	107.00
C6—C1—C29	115.0 (4)	C11—C12—H12A	109.00
O1—C2—C3	118.6 (5)	C11—C12—H12B	109.00
C1—C2—C3	120.7 (5)	C13—C12—H12A	109.00
O1—C2—C1	120.7 (5)	C13—C12—H12B	109.00
C2—C3—C4	113.9 (4)	H12A—C12—H12B	108.00
C3—C4—C5	113.4 (4)	C14—C15—H15A	111.00
C4—C5—C6	106.4 (3)	C14—C15—H15B	111.00
C4—C5—C10	110.0 (3)	C16—C15—H15A	111.00
C6—C5—C10	108.6 (3)	C16—C15—H15B	111.00
C6—C5—C28	115.1 (4)	H15A—C15—H15B	109.00
C10—C5—C28	108.1 (4)	C15—C16—H16A	110.00
C4—C5—C28	108.6 (4)	C15—C16—H16B	110.00
C1—C6—C5	118.0 (3)	C17—C16—H16A	110.00
C5—C6—C7	110.6 (3)	C17—C16—H16B	110.00
C1—C6—C7	112.6 (4)	H16A—C16—H16B	108.00
C6—C7—C8	109.8 (4)	C13—C17—H17	107.00
C7—C8—C9	117.5 (3)	C16—C17—H17	107.00
C8—C9—C14	117.5 (3)	C18—C17—H17	107.00
C10—C9—C14	119.7 (3)	C17—C18—H18	108.00
C8—C9—C10	122.6 (3)	C19—C18—H18	108.00
C5—C10—C11	117.9 (3)	C25—C18—H18	108.00
C9—C10—C11	119.8 (3)	C18—C19—H19A	108.00
C5—C10—C9	120.6 (4)	C18—C19—H19B	108.00
C10—C11—C12	119.6 (3)	C20—C19—H19A	108.00
C11—C12—C13	112.6 (3)	C20—C19—H19B	108.00
C12—C13—C17	119.2 (3)	H19A—C19—H19B	107.00
C12—C13—C26	109.1 (3)	C19—C20—H20A	109.00
C14—C13—C17	101.1 (3)	C19—C20—H20B	109.00
C14—C13—C26	111.1 (3)	C21—C20—H20A	109.00
C17—C13—C26	108.9 (3)	C21—C20—H20B	109.00
C12—C13—C14	107.2 (3)	H20A—C20—H20B	108.00
C9—C14—C13	111.9 (3)	C20—C21—H21	115.00
C9—C14—C27	105.8 (3)	C22—C21—H21	115.00
C13—C14—C15	101.5 (3)	C22—C24—H24A	109.00
C13—C14—C27	113.3 (3)	C22—C24—H24B	109.00
C15—C14—C27	107.0 (3)	C22—C24—H24C	109.00
C9—C14—C15	117.6 (3)	H24A—C24—H24B	109.00
C14—C15—C16	104.3 (3)	H24A—C24—H24C	110.00
C15—C16—C17	107.6 (3)	H24B—C24—H24C	109.00
C13—C17—C18	120.7 (3)	C18—C25—H25A	109.00
C16—C17—C18	112.2 (3)	C18—C25—H25B	109.00
C13—C17—C16	102.3 (3)	C18—C25—H25C	109.00
C17—C18—C19	108.4 (3)	H25A—C25—H25B	109.00
C17—C18—C25	114.0 (3)	H25A—C25—H25C	110.00
C19—C18—C25	110.6 (4)	H25B—C25—H25C	109.00
C18—C19—C20	115.3 (3)	C13—C26—H26A	109.00
C19—C20—C21	111.8 (4)	C13—C26—H26B	109.00
C20—C21—C22	131.0 (4)	C13—C26—H26C	109.00
C21—C22—C24	121.0 (4)	H26A—C26—H26B	110.00
C23—C22—C24	115.0 (3)	H26A—C26—H26C	109.00
C21—C22—C23	123.9 (4)	H26B—C26—H26C	110.00
O2—C23—C22	120.1 (4)	C14—C27—H27A	109.00
O3—C23—C22	118.0 (4)	C14—C27—H27B	109.00
O2—C23—O3	121.9 (4)	C14—C27—H27C	109.00
C2—C3—H3A	109.00	H27A—C27—H27B	110.00
C2—C3—H3B	109.00	H27A—C27—H27C	109.00
C4—C3—H3A	109.00	H27B—C27—H27C	110.00
C4—C3—H3B	109.00	C5—C28—H28A	109.00
H3A—C3—H3B	108.00	C5—C28—H28B	109.00
C3—C4—H4A	109.00	C5—C28—H28C	109.00
C3—C4—H4B	109.00	H28A—C28—H28B	109.00
C5—C4—H4A	109.00	H28A—C28—H28C	109.00
C5—C4—H4B	109.00	H28B—C28—H28C	110.00
H4A—C4—H4B	108.00	C1—C29—H29A	109.00
C1—C6—H6	105.00	C1—C29—H29B	109.00
C5—C6—H6	105.00	C1—C29—H29C	109.00
C7—C6—H6	105.00	H29A—C29—H29B	110.00
C6—C7—H7A	110.00	H29A—C29—H29C	109.00
C6—C7—H7B	110.00	H29B—C29—H29C	109.00
C8—C7—H7A	110.00	C1—C30—H30A	109.00
C8—C7—H7B	110.00	C1—C30—H30B	109.00
H7A—C7—H7B	108.00	C1—C30—H30C	109.00
C7—C8—H8A	108.00	H30A—C30—H30B	109.00
C7—C8—H8B	108.00	H30A—C30—H30C	110.00
C9—C8—H8A	108.00	H30B—C30—H30C	109.00
C9—C8—H8B	108.00		
			
C6—C1—C2—O1	149.9 (5)	C10—C9—C14—C15	−148.9 (4)
C6—C1—C2—C3	−32.5 (6)	C10—C9—C14—C27	91.7 (5)
C29—C1—C2—O1	−84.9 (6)	C5—C10—C11—C12	176.1 (4)
C29—C1—C2—C3	92.7 (6)	C9—C10—C11—C12	11.1 (7)
C30—C1—C2—O1	31.6 (7)	C10—C11—C12—C13	19.5 (7)
C30—C1—C2—C3	−150.8 (5)	C11—C12—C13—C14	−52.8 (5)
C2—C1—C6—C5	40.4 (5)	C11—C12—C13—C17	−166.6 (4)
C2—C1—C6—C7	171.2 (4)	C11—C12—C13—C26	67.6 (5)
C29—C1—C6—C5	−79.7 (5)	C12—C13—C14—C9	59.9 (4)
C29—C1—C6—C7	51.1 (5)	C12—C13—C14—C15	−173.9 (3)
C30—C1—C6—C5	158.2 (4)	C12—C13—C14—C27	−59.6 (4)
C30—C1—C6—C7	−71.0 (5)	C17—C13—C14—C9	−174.6 (3)
O1—C2—C3—C4	−142.5 (6)	C17—C13—C14—C15	−48.4 (4)
C1—C2—C3—C4	39.9 (8)	C17—C13—C14—C27	65.9 (4)
C2—C3—C4—C5	−52.6 (7)	C26—C13—C14—C9	−59.2 (4)
C3—C4—C5—C6	57.3 (5)	C26—C13—C14—C15	67.0 (4)
C3—C4—C5—C10	174.7 (4)	C26—C13—C14—C27	−178.7 (3)
C3—C4—C5—C28	−67.1 (5)	C12—C13—C17—C16	156.9 (4)
C4—C5—C6—C1	−52.9 (5)	C12—C13—C17—C18	−77.8 (5)
C4—C5—C6—C7	175.4 (3)	C14—C13—C17—C16	39.9 (4)
C10—C5—C6—C1	−171.3 (4)	C14—C13—C17—C18	165.2 (3)
C10—C5—C6—C7	57.1 (4)	C26—C13—C17—C16	−77.1 (4)
C28—C5—C6—C1	67.4 (5)	C26—C13—C17—C18	48.2 (5)
C28—C5—C6—C7	−64.2 (4)	C9—C14—C15—C16	159.8 (4)
C4—C5—C10—C9	−145.8 (4)	C13—C14—C15—C16	37.5 (4)
C4—C5—C10—C11	49.4 (5)	C27—C14—C15—C16	−81.5 (4)
C6—C5—C10—C9	−29.7 (6)	C14—C15—C16—C17	−12.7 (5)
C6—C5—C10—C11	165.4 (4)	C15—C16—C17—C13	−17.3 (5)
C28—C5—C10—C9	95.8 (5)	C15—C16—C17—C18	−148.0 (4)
C28—C5—C10—C11	−69.1 (6)	C13—C17—C18—C19	178.4 (3)
C1—C6—C7—C8	165.3 (4)	C13—C17—C18—C25	54.8 (5)
C5—C6—C7—C8	−60.3 (5)	C16—C17—C18—C19	−61.0 (4)
C6—C7—C8—C9	35.0 (6)	C16—C17—C18—C25	175.4 (4)
C7—C8—C9—C10	−7.9 (7)	C17—C18—C19—C20	172.5 (4)
C7—C8—C9—C14	166.7 (4)	C25—C18—C19—C20	−61.9 (5)
C8—C9—C10—C5	5.7 (7)	C18—C19—C20—C21	−176.2 (4)
C8—C9—C10—C11	170.3 (4)	C19—C20—C21—C22	109.0 (5)
C14—C9—C10—C5	−168.8 (4)	C20—C21—C22—C23	−1.6 (7)
C14—C9—C10—C11	−4.2 (7)	C20—C21—C22—C24	176.6 (4)
C8—C9—C14—C13	153.2 (4)	C21—C22—C23—O2	−9.1 (7)
C8—C9—C14—C15	36.4 (5)	C21—C22—C23—O3	171.7 (4)
C8—C9—C14—C27	−83.0 (4)	C24—C22—C23—O2	172.6 (4)
C10—C9—C14—C13	−32.1 (5)	C24—C22—C23—O3	−6.7 (6)

Symmetry codes: (i) *y*, *x*, −*z*; (ii) *y*, *x*, −*z*+1; (iii) −*y*+1/2, *x*+1/2, *z*−1/4; (iv) −*y*+1/2, *x*−1/2, *z*−1/4; (v) *x*+1/2, −*y*+1/2, −*z*+1/4; (vi) *y*+1/2, −*x*+1/2, *z*+1/4; (vii) *y*−1/2, −*x*+1/2, *z*+1/4; (viii) −*x*+1/2, *y*+1/2, −*z*+3/4; (ix) −*y*, −*x*, −*z*+1/2; (x) *x*−1/2, −*y*+1/2, −*z*+1/4; (xi) −*x*+1/2, *y*−1/2, −*z*+3/4.

### Hydrogen-bond geometry (Å, °)

**Table:**

*D*—H···*A*	*D*—H	H···*A*	*D*···*A*	*D*—H···*A*
O2—H2···O3^i^	0.82	1.85	2.645 (7)	162
C20—H20A···O2	0.97	2.21	2.873 (7)	125

Symmetry codes: (i) *y*, *x*, −*z*.
